# Trends in infections detected in women with cervicitis over a decade

**DOI:** 10.3389/frph.2025.1539186

**Published:** 2025-02-03

**Authors:** Lenka A. Vodstrcil, Erica L. Plummer, Thuy Vy Nguyen, Christopher K. Fairley, Eric P. F. Chow, Tiffany R. Phillips, Catriona S. Bradshaw

**Affiliations:** ^1^ School of Translational Medicine, Monash University, Melbourne, VIC, Australia; ^2^ Melbourne Sexual Health Centre, Alfred Health, Melbourne, VIC, Australia; ^3^ Centre for Epidemiology and Biostatistics, Melbourne School of Population and Global Health, The University of Melbourne, Melbourne, VIC, Australia

**Keywords:** cervicitis, sexually transmitted infections, *Mycoplasma genitalium*, bacterial vaginosis, *Chlamydia trachomatis*, *Neisseria gonorrhoeae*

## Abstract

**Objectives:**

There is a growing body of evidence that in the absence of *Chlamydia trachomatis* and/or *Neisseria gonorrhoeae, Mycoplasma genitalium* and bacterial vaginosis (BV) are associated with cervicitis. We aimed to describe infections detected among cervicitis cases over a decade and establish how commonly *M. genitalium* and BV were detected among non-chlamydial/non-gonococcal cases to inform testing and treatment practices.

**Methods:**

We conducted a retrospective case-series to determine the number of cervicitis cases diagnosed with genital infections (*C. trachomatis*, *N. gonorrhoeae, M. genitalium* and BV) among women attending the largest public sexual health service in Australia from 2011 to 2021. We determined the proportion of cervicitis cases with one or more genital infections detected, and trends in testing and detection of each infection over time.

**Results:**

Over a decade 813 cervicitis cases were diagnosed; 421 (52%, 95%CI: 48%–55%) had no infection detected; 226/729 (31%, 95%CI: 28%–35%) had BV, 163/809 (20%, 95%CI: 17%–23%) *C. trachomatis*, 48/747 (6%, 95%CI: 5%–8%) *M. genitalium*, and 13/793 (2%, 95%CI: 1%–3%) *N. gonorrhoeae*. Of the 665 (82%) cases tested for all four infections, 268 (40%) had one infection and 73 (11%) had >1 infection detected. Of the 517/665 (78%) non-chlamydial/non-gonococcal cases*,* 164 (32%) had BV and 16 (3%) had *M. genitalium* as the sole infections detected; a further 13 cases (3%) were co-infected with BV and *M. genitalium*. The proportion of cases tested for BV (90%) did not change overtime, but detection increased from 32% to 45% (P_trend_ < 0.001). The proportion of cases tested for *M. genitalium* increased from 84% in 2011 to 96% in 2019 (P_trend_ = 0.006), with *M. genitalium*-detection in cervicitis increasing from 3% to 7% (P_trend_ = 0.046).

**Conclusions:**

In our study population, chlamydia or gonorrhoea were not detected in ∼75% of cervicitis cases; 1 in 3 of these cases had BV and/or *M. genitalium*, and both increased in prevalence over time. These data highlight the need for clinicians to consider BV and *M. genitalium* when assessing and managing cervicitis.

## Introduction

Cervicitis is defined by the presence of mucopurulent discharge and/or cervical friability at the endocervical os (1, 2). Cervicitis is associated with HIV transmission (3) and is a potential precursor of upper genital tract infection, resulting in pelvic inflammatory disease (PID) and obstetric sequelae (4, 5). Identification and effective treatment of cervicitis is important to relieve symptoms and prevent sequelae.

*Chlamydia trachomatis* and *Neisseria gonorrhoeae* have traditionally been considered the most common causes of cervicitis. Other etiologic pathogens less commonly attributed to cervicitis include *Trichomonas vaginalis* and Herpes Simplex Virus (HSV) (2, 6). In the absence of these pathogens, *Mycoplasma genitalium* and the common vaginal dysbiosis, bacterial vaginosis (BV), are emerging as other infections associated with cervicitis. *M. genitalium* is associated with cervicitis by meta-analysis [pooled odds ratio (OR) = 1.66, 95%CI: 1.35–2.04] (7), and in a recent case-control study of >1,300 women in Melbourne, Australia, *M. genitalium* was associated with mucopurulent cervicitis on examination (adjusted OR = 4.38, 95% CI: 1.69–11.33, *p* = 0.002) (8). Limited studies have also shown that BV is associated with cervicitis (9–11). A secondary analysis of the same Australian case-control study, which utilised samples from 65 STI-negative cases with cervicitis and 128 STI-negative asymptomatic women, found that STI-negative cervicitis cases were five times more likely to have BV compared to STI-negative asymptomatic controls (12). Cases were also more likely than controls to have a *Lactobacillus*-deficient non-optimal microbiota and an increased abundance of four BV-associated bacteria (*Gardnerella, Fannyhessea vaginae, Prevotella bivia, Dialister micraerophilus*) (12).

Despite this evidence, the relative contribution of *M. genitalium* and BV to the burden of cervicitis is poorly understood, leading to inconsistency in testing recommendations across international guidelines (2, 13–15). The majority of guidelines list chlamydia and gonorrhoea as the main causes of cervicitis and recommend routine testing, but these infections are detected in <30%–40% of cases (2, 16–18), leaving a high proportion of cases with no clear cause (Supplementary Table 1).

We aimed to describe the proportion of cervicitis cases with *C. trachomatis*, *N. gonorrhoeae, M. genitalium* and/or BV, and to determine how commonly *M. genitalium* and BV were detected among women with non-chlamydial, non-gonococcal cervicitis in order to inform contemporary testing and management practices.

## Methods

This retrospective case-series incorporated women with cervicitis who attended Melbourne Sexual Health Centre (MSHC) from 1st March 2011 to 1st March 2021. Ethical approval was provided by the Alfred Hospital Ethics Committee (ID: 718/20).

Women presenting with vaginal, pelvic and/or abdominal symptoms underwent a speculum and bimanual examination, STI testing, and microscopy of vaginal secretions. Cervicitis was defined as the presence of mucopurulent discharge at the cervical os and/or cervical ectopy or a friable cervix with easily induced bleeding (13). Clinical signs, symptoms, diagnoses, and test results were documented.

### Testing for genital infections

The details of laboratory testing performed for all STIs and BV are as previously reported (19–21), with extended information supplied in Supplementary Material*—Extended Methodology*. Briefly, *C. trachomatis* was universally screened for. Before March 2015, *N. gonorrhoeae* was tested for in symptomatic women, sex workers, and sexual contacts, and subsequently, attendees were universally screened for *N. gonorrhoeae*. Indications for *M. genitalium* testing included cervicitis, pelvic pain, post-coital bleeding, test-of-cure, and sexual contact of infection. Throughout the study period, vaginal microscopy (Gram stain and wet preparation) was performed in all women presenting with abnormal vaginal discharge, itch, vaginal malodour, pelvic pain, cervicitis, and post-coital bleeding. BV was assessed and diagnosed using a combination of both Amsel and Nugent criteria (22, 23). Selective testing for *T. vaginalis* and genital HSV occurred among cervicitis cases based on the presence of relevant clinical signs and indications (19, 24).

### Data extraction

Epidemiological and clinical data were extracted for all individuals >18 years with a diagnosis of “cervicitis” documented. File review captured additional relevant data from an adjacent clinical visit within 4 weeks of the cervicitis diagnosis (i.e., if a case had chlamydia in the week preceding the cervicitis diagnosis, this recent infection was attributed to the case). Among those with >1 cervicitis diagnosis within 6 months (*n* = 16), either the first cervicitis diagnosis or the diagnosis with the most complete data was included. For the purposes of this study, each 12-month period incorporated March of 1 year to March of the subsequent year (i.e., ″2011″ comprises data from 1st March 2011 to 1st March 2012).

### Statistical analysis

All proportions, 95% confidence intervals (CIs; using exact binomial method), and statistical analyses were conducted using Stata (v14, StataCorp LP, College Station, TX, USA).

We extracted the number of cervicitis cases, and calculated the number tested for, and the proportion with, *C. trachomatis, N. gonorrhoeae, M. genitalium*, BV, *T. vaginalis* and/or HSV. Utilising recently published data from our service for *C. trachomatis* and *N. gonorrhoeae* (25), supplemented with additional data for *M. genitalium* and BV, we next calculated the proportion of first-time MSHC attendees tested for, and with each genital infection (including BV), during an equivalent period. This approach enabled an estimate of the annual change in cervicitis diagnoses to be generated, however was limited, as not all women were examined for cervicitis. Due to differing indications for testing/screening, and because asymptomatic cervicitis cases were potentially missed, the two groups were not statistically compared.

The following analyses were restricted to visits prior to 1st March 2020 (i.e., includes 2011–2019, as defined above), after which there was a significant impact of COVID-19 lockdowns on MSHC attendance (26). Poisson regression was used to examine changes in the number of first-time MSHC attendees and cervicitis cases over time, and the chi-square trend test applied to establish trends in the proportion of attendees diagnosed with cervicitis (excluding repeat visits in the same year to ensure that recurrent/persistent infections did not influence estimates). Trends in the proportion tested for each infection and positive for each infection were then assessed separately among cervicitis cases and the MSHC population (25).

## Results

A total of 840 cervicitis diagnoses were recorded over the study period; 27 cases were excluded as individuals were ≤18 years of age (*n* = 11) or they had a repeat cervicitis diagnosis *within* 6 months (*n* = 16). The remaining 813 cases were from 805 attendees with at least one diagnosis of cervicitis >6 months apart. None of the identified cases were women living with HIV.

### Testing patterns and genital infections among cervicitis cases

Table 1 outlines the number of cervicitis cases tested for each genital infection and the proportion with each infection. Of the 813 cases, 421 (51.8%, 95%CI:48.3%–55.3%) had no infections identified; two of the 421 did not have a *C. trachomatis* test documented, 19 did not have a *N. gonorrhoeae* test documented, 64 did not have an *M. genitalium* test documented and 55 did not have Amsel and/or Nugent criteria documented (i.e., no BV result documented). Of those with an infection identified, 392 (48.2%, 95%CI: 44.7%–51.7%) had at least one infection detected; 90.3% of whom had one infection, and 9.7% ≥2 infections detected.

**Table 1:** Detection of *Chlamydia trachomatis, Neisseria gonorrhoeae, Mycoplasma genitalium*, bacterial vaginosis, *trichomonas vaginalis* and herpes simplex virus among women with cervicitis 2011–2020, and among all women attending for their first visit to MSHC 2011–2019.

	No. of women with cervicitis who were tested for, and diagnosed with, each infection *n*, *N*	% of cervicitis cases with each infection % (95% CI)	No. of all female attendees tested for, and diagnosed with, each infection at their first visit to MSHC *n*/*N*	% of all female attendees with each infection at their first visit to MSHC % (95% CI)
*C. trachomatis*				
Negative	646, 809	79.85 (76.9–82.6)	30,809/33,833	91.06 (90.8–91.4)
Positive	163, 809	20.15 (17.4–23.1)	3,024/33,833	8.94 (8.6–9.3)
Not tested^a^	4/813	0.49 (0.1–1.3)	2,314/36,147	6.40 (6.5–6.7)
*N. gonorrhoeae*				
Negative	780/793	98.36 (97.2–99.1)	22,139/22,398	98.84 (98.7–99.0)
Positive	13/793	1.64 (0.9–2.8)	259/22,398	1.16 (1.0–1.3)
Not tested^a^	20/813	2.46 (1.5–3.8)	13,749/36,147	38.03 (37.5–38.5)^b^
*M. genitalium*				
Negative	699/747	93.57 (91.6–95.2)	4,232/4,728	89.51 (88.7–90.4)
Positive	48/747	6.43 (4.8–8.4)	496/4,728	10.49 (9.6–11.4)
Not tested^a^	66/813	8.12 (6.3–10.2)	31,436/36,147	86.97 (86.6–87.3)
Bacterial vaginosis				
Negative	503/729	69.00 (65.5–72.3)	8,783/12,128	72.42 (71.6–73.2)
Positive	226/729	31.00 (27.7–34.5)	3,345/12,128	27.58 (26.8–28.4)
Not tested^a^	84/813	10.33 (8.3–12.6)	24,036/36,147	66.50 (66.0–67.0)
*T. vaginalis*				
Negative	129/135	95.56 (90.6–98.4)	8,539/8,589	99.42 (99.2–99.6)
Positive	6/135	4.44 (1.6–9.4)	50/8,589	0.58 (0.4–0.8)
Not tested^a^	678/813	83.39 (80.7–85.9)	27,558/36,147	76.24 (75.8–76.7)
Herpes simplex virus				
Negative	64/88	72.72 (62.2–81.7)	34,973/36,147	96.75 (96.6–96.9)
Positive	24/88	27.27 (18.3–37.8)	1,174/36,147	0.03 (3.1–3.4)
*HSV-1*	13/24		–	–
*HSV-2*	7/24		–	–
*Untyped*	4/24		–	–
Not tested^a^	725/813	89.18 (86.8–91.2)	–	–

N.B. Cells have been left empty (–) if data was unavailable or because testing was specific to those presenting with clinically relevant symptoms and/or signs. There was no infectious cause detected in 421 women diagnosed with cervicitis.

^a^ “Not tested” data reflect the number of women who were not tested, and the proportion not tested, firstly for women with cervicitis and secondly, for women attending for their first visit at MSHC.

^b^ The large proportion not tested reflects the period prior to March 2015, when indications for *N. gonorrhoeae* testing only included cervicitis, pelvic inflammatory disease (PID), post-coital bleeding, test-of-cure, sex worker, and sexual contact of *N. gonorrhoeae* infection. During this period, *N. gonorrhoeae* testing involved culture (swabs plated at the bedside onto a modified Thayer-Martin medium) and a cervical smear that was Gram stained and underwent immediate microscopy. From March 2015, all women were *universally screened* for *N. gonorrhoeae* using the multiplex AC2-assay (Hologic, combined with chlamydia).

Of the 809/813 (99.5%, 95%CI: 98.7%–99.9%) cervicitis cases tested for *C. trachomatis,* 163 (20.1%, 95%CI: 17.4%–23.1%) had *C. trachomatis.* Of the 793/813 cases (97.5%, 95%CI: 96.2%–98.5%) tested for *N. gonorrhoeae,* only 13 (1.6%, 95%CI: 0.9%–2.8%) had *N. gonorrhoeae* detected. Of the 747/813 (91.9%, 95%CI: 89.8%–93.7%) cervicitis cases tested for *M. genitalium*, 48 (6.4%, 95%CI: 4.8%–8.4%) had *M. genitalium* detected. 729 (89.7%, 95%CI: 87.4%–91.7%) cases were assessed for BV, and 226 (31.0%, 95%CI: 27.7%–34.5%) had BV diagnosed.

Select testing for *T. vaginalis* and HSV was based on clinical indications (as per clinical guidelines). Of the 135/813 (16.6% 95%CI: 14.1%–19.3%) cases selectively tested for *T. vaginalis*, six (4.4%, 95%CI: 1.6%–9.4%) had *T. vaginalis* detected. Additionally, 88/813 (10.8%, 95%CI: 8.8%–13.2%) cervicitis cases were selectively tested for HSV; 24 (27.3%, 95%CI: 18.3%–37.8%) had HSV detected.

**Figure 1:**
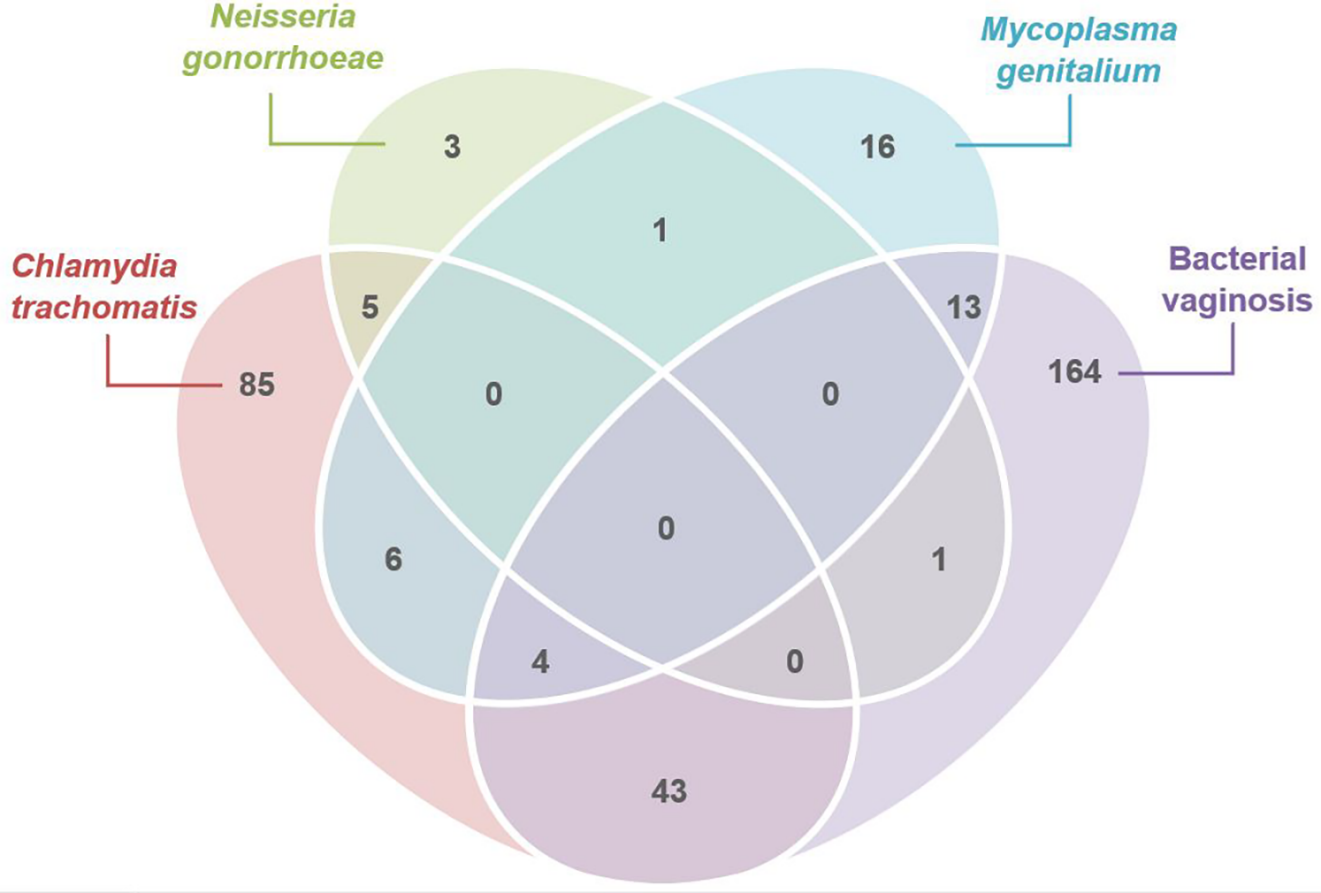
Venn diagram illustrating the number of women with cervicitis attending MSHC who were tested for *C. trachomatis, M. genitalium, N. gonorrhoeae* and bacterial vaginosis (BV) (*N* = 665), and who had either mono-infections detected (*n* = 268), or who were co-infected (*n* = 73) with one or more infections (2011–2020). Of those without *C. trachomatis* or *N. gonorrhoeae* (*n* = 517)*,* 193 women with cervicitis had either *M. genitalium* (*n* = 16), bacterial vaginosis (*n* = 164) or both *M. genitalium* and BV detected (*n* = 13).

We next looked at mono- and co-infections among cases tested for *C. trachomatis, N. gonorrhoeae, M. genitalium* and BV and determined the proportion of non-chlamydial, non-gonococcal cervicitis with *M. genitalium* and/or BV. Of the *N* = 665 (81.8%) cervicitis cases tested for all four of these infections, 268 (40.3%) had a single infection detected, and 73 (11.0%) had more than one infection detected, Figure 1. *C. trachomatis* or *N. gonorrhoeae* accounted for 22.3% (*n* = 148) of infection in cases of cervicitis. The most common co-infection among these cases was *C. trachomatis* with BV (*n* = 43/73; 58.9% of coinfections). Of 517 cases (77.7%) with non-chlamydial, non-gonococcal cervicitis, BV and/or *M. genitalium* was the sole infection detected in 193/517 (37.3%) of cases; BV was the only infection detected in 164/517 (31.7%) cervicitis cases, and *M. genitalium* was the sole infection detected in 16/517 (3.1%) cervicitis cases. Thirteen cases (2.5%) were co-infected with BV and *M. genitalium*.

### Trends in cervicitis and genital infections

The following analyses were restricted to a woman's first-ever cervicitis diagnosis or visit to MSHC during 2011–2019, as described above.

**Figure 2:**
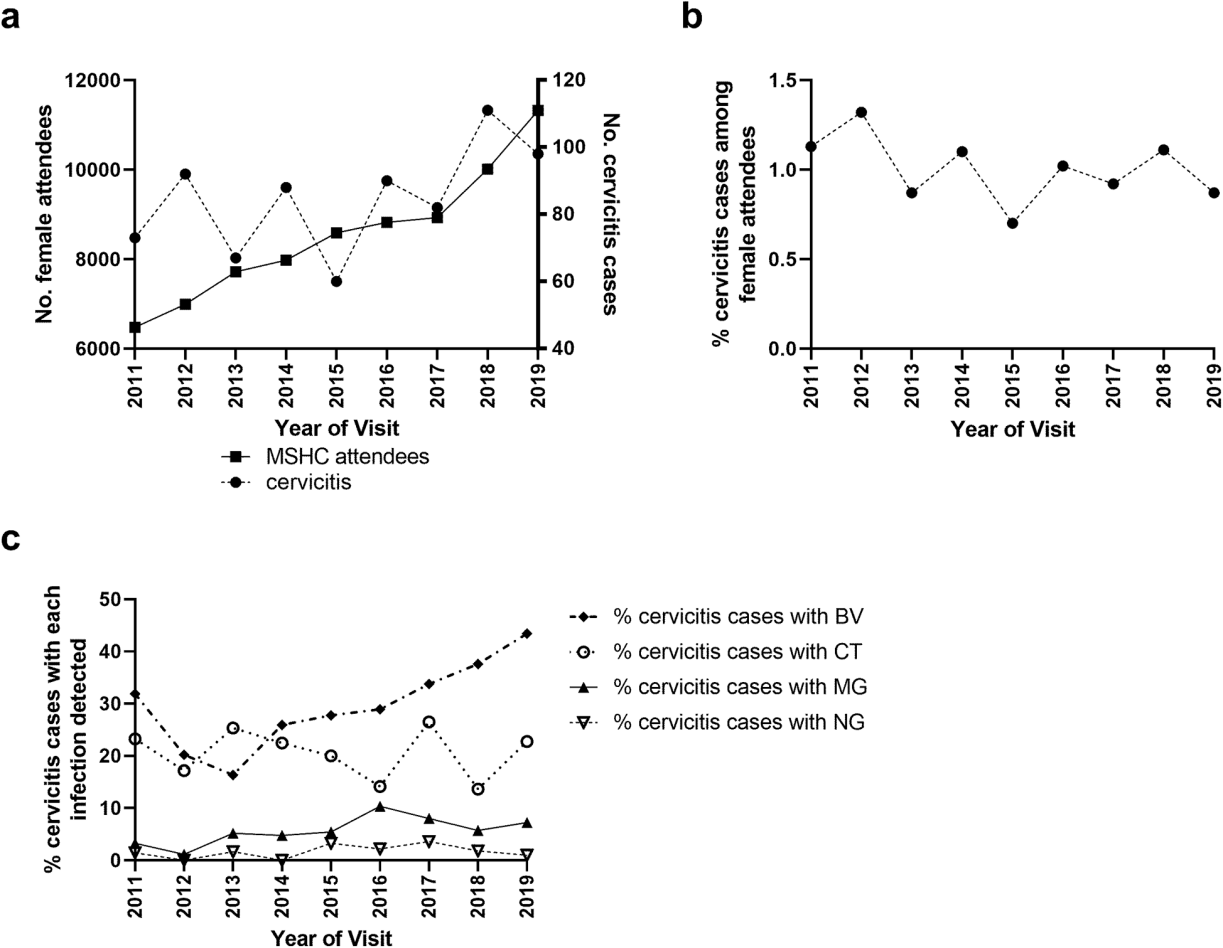
Distribution of **(a)** the number of women attending MSHC for the first-time and the number of cervicitis cases over time (2011–2019) and **(b)** the proportion of unique women who had a cervicitis diagnosis overtime. The number of first-ever cervicitis diagnoses of women attending the service was divided by the total number of unique attendees per annum. **(c)** The proportion of cervicitis cases diagnosed with each of the four infections (*C. trachomatis* [CT], *N. gonorrhoeae* [NG], *M. genitalium* [MG] and bacterial vaginosis [BV]) per annum. Only the first cervicitis diagnosis for each woman was included within temporal analyses.

The number of unique MSHC attendees significantly increased over time (*p* < 0.001), from 6,475 in 2011 to 11,323 in 2019, and the number of cervicitis cases increased from 60 in 2015 to 98 in 2019 (*p* = 0.006), Figure 2a. The cases of cervicitis as a proportion of MSHC attendees was low and ranged from 0.9% to 1.1% and remained stable over time (P_trend_ = 0.080), Figure 2b.

We next calculated the trends in the proportion of MSHC attendees from 2011 to 2019 who (i) were tested for and (ii) tested positive for each of the four genital infections (Figure 2c), and then repeated these analyses for cervicitis cases over the same period. The proportion of MSHC attendees who were tested for *C. trachomatis* was high and increased from 91% in 2011 to 94% in 2019 (P_trend_ < 0.001), with no change in positivity over time (P_trend_ = 0.168; Supplementary Figure 1). Among cervicitis cases, *C. trachomatis* testing was consistently high (range = 98%–100%; P_trend_ = 0.705), and *C. trachomatis* positivity in cervicitis cases also did not change over time (range = 14%–25%, P_trend_ = 0.467). The proportion of all attendees tested for *N. gonorrhoeae* increased from 49% in 2011 to 95% in 2019 (P_trend_ < 0.001), corresponding to a change from selective testing to universal screening in March 2015. However, the proportion of all attendees who tested positive for *N. gonorrhoeae* did not change over time (range = 1%–2%, P_trend_ = 0.140; Supplementary Figure 1). Among cervicitis cases, the proportion tested for *N. gonorrhoeae* increased from 96% in 2011 to 100% in 2019 (P_trend_ < 0.001), but again positivity in cervicitis cases was stable (range = 0%–4%, P_trend_ = 0.367).

**Figure 3:**
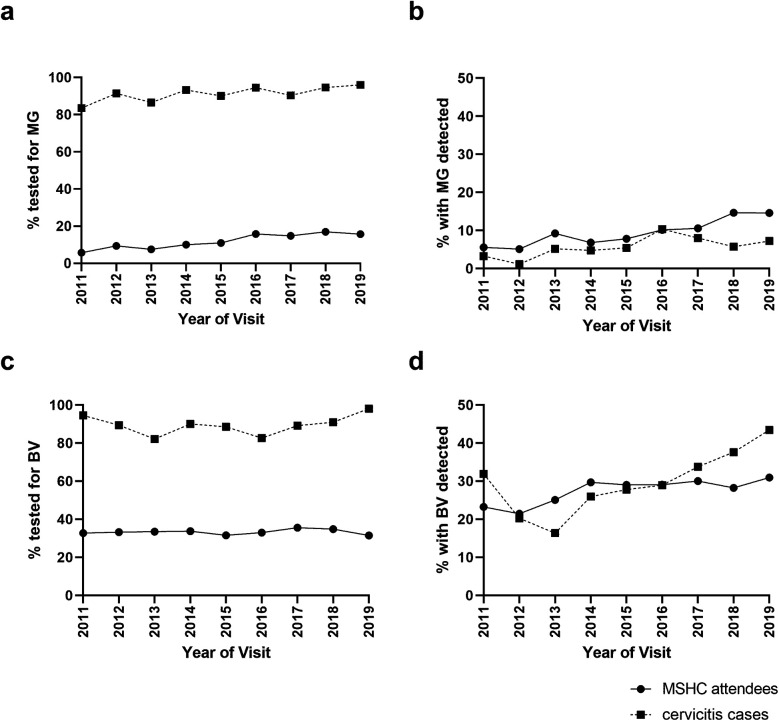
*Mycoplasma genitalium* (MG) and bacterial vaginosis (BV) testing and positivity among women with cervicitis and all women attending MSHC overtime. The proportion of cervicitis cases or all MSHC first-ever attendees who were **(a)** tested for *M. genitalium,* **(b)** had *M. genitalium* detected, **(c)** tested for BV or **(d)** had BV detected at MSHC between 2011 and 2019 was calculated.

The proportion of all attendees tested for *M. genitalium* significantly increased from 6% in 2011 to 16% in 2019 (P_trend_ < 0.001, Figure 3a), and positivity also increased from 6% in 2011 to 15% in 2019 (P_trend_ < 0.001), Figure 3b. Among women with cervicitis, the proportion tested for *M. genitalium* increased from 84% in 2011 to 96% in 2019 (P_trend_ = 0.006), and positivity increased from 3% in 2011 to 7% in 2019 (P_trend_ = 0.046). The proportion of women attending MSHC tested for BV at their first visit decreased slightly but significantly from 33% in 2011 to 31% in 2019 (P_trend_ < 0.001, Figure 3c), however the proportion with BV detected increased from 23% in 2011 to 31% in 2019 (P_trend_ < 0.001), Figure 3d. Although testing for BV in cervicitis was high (average = 89%) and ranged from 82% to 98% in 2019, it did not significantly change over time (P_trend_ = 0.359), however, BV positivity in cervicitis cases significantly increased from 32% in 2011 to 45% in 2019 (P_trend_ < 0.001).

## Discussion

This retrospective study examined infections detected among women with cervicitis attending the largest public sexual health centre in Australia over a decade. This study was not designed to determine causality but to establish the proportion of cervicitis cases with *C. trachomatis*, *N. gonorrhoeae, M. genitalium* and/or BV, and to estimate how commonly *M. genitalium* and BV were detected among women with non-chlamydial, non-gonococcal cervicitis. As expected, a substantial proportion of cervicitis cases tested positive for *C. trachomatis*, with no significant change in the proportion of cases attributable to *C. trachomatis* over time. In contrast, *N. gonorrhoeae* was rarely detected among cervicitis cases. Collectively these two infections were only detected in 22% of cases tested for all four genital infections. In women with non-chlamydial non-gonococcal cervicitis, BV and *M. genitalium* were diagnosed in a third of cases, with BV the most common sole diagnosis made. Furthermore, despite no increase in testing, the number of BV diagnoses among cervicitis cases significantly increased throughout the study period. *M. genitalium* was the third most common infection found in cervicitis cases, and *M. genitalium* detection among cases and the clinic population both significantly increased over time from 3%–7% to 6%–15%, respectively. Although these data cannot establish causation, this study supports recent findings on the association between both BV (and BV-associated bacteria) and *M. genitalium* in case-control studies and informs contemporary clinical practice.

There are a limited number of studies that investigate the relationship between BV/BV-associated bacteria and cervicitis (9–12, 27, 28). The most recent evidence is from a case-control study of 65 STI-negative cases with cervicitis and 128 STI-negative asymptomatic women attending for STI-screening at MSHC. Cases with STI-negative cervicitis were 5 times more likely to have BV than STI-negative asymptomatic controls and in vaginal microbiota analyses, there was an increased abundance of specific BV-associated bacteria in cases compared to controls (12). This evidence builds on prior studies showing an association between BV (and BV-associated bacteria) and cervicitis. One US study reported that 67/423 (15%) BV cases had cervicitis, and of these, most (87%) had no other pathogen detected (11). In a small Seattle cohort, of which 14/210 had cervicitis, the BV-associated species, *Mageeibacillus indolicus*, was significantly associated with cervicitis, although this finding was not replicated among the comparator Kenyan cohort (28). Among a Chinese cohort, using qPCR *Prevotella* was more abundant in BV-positive cervicitis cases vs. people without cervicitis or BV (27). Emerging evidence also suggests that BV-associated bacteria account for a significant proportion of PID cases. One large prospective study of 2,956 women found BV was associated with incident PID, after adjusting for concurrent STIs (29). Two recent reviews reported that <50% of women with PID had chlamydial or gonococcal infections, while *M. genitalium* and detection of BV/BV-associated bacteria (*Sneathia* spp.*, Atopobium vaginae, Megasphaera* spp.) accounted for a substantial fraction of PID cases (30, 31). In support of this, the addition of metronidazole to empiric PID treatment improved clinical outcomes for people with PID (32). Furthermore, the addition of metronidazole to cervicitis treatment improved cervicitis resolution, and people with BV were more likely to experience resolution (10). Clearly, there is a need for prospective research to improve our understanding of causal relationships between BV or specific BV-associated bacteria and the genital syndromes of cervicitis and PID, although current evidence supports consideration of BV in diagnostic recommendations.

As the third most common infection detected, *M. genitalium* positivity among cervicitis cases was 6% overall and increased over time, although this may reflect increased testing. Throughout the study period two different PCR assays were used with similar sensitivity, but lower sensitivity than a transcription-mediated amplification (TMA) assay (33). As *M. genitalium* is often a low load infection it is possible that positivity may have been under-reported, but even based on an additional 25%–30% of infections being detected by TMA, this would not increase the overall proportion of cases to more than 8%. A recent study at our service showed *M. genitalium* to be associated with a four-fold increased odds of cervicitis (8), in line with other publications (7, 34). Importantly however, *M. genitalium* was often co-detected with other infections, especially BV. BV may enhance an individual's risk of *M. genitalium* infection (35), although the importance of co-infections to an individual's susceptibility to cervicitis remains unknown.

Over the study period, *C. trachomatis* remained a significant contributor to cervicitis; it was detected in 1 in 5 cervicitis cases, which is within the previously reported range (11%–50%) (1, 36, 37), but greater than a similar Sydney-based population (6%) (38). Although the number of attendees to MSHC increased over time, the proportion of cervicitis cases with *C. trachomatis* was stable. This finding may be explained by a relative decrease in the contribution of *C. trachomatis* to cervicitis in comparison to other emerging infections, which has also been reported in relation to PID (30, 31). The low prevalence of *N. gonorrhoeae* was expected and is in line with prevalence in urban Australian populations (39). It is possible that increased screening for chlamydia and gonorrhoea, and practices such as expedited treatment, have reduced the risk of women developing more severe symptoms/syndromes including cervicitis. It is important to note that in some populations, *N. gonorrhoeae* positivity is higher and may have a greater contribution to cervicitis, highlighting the need for contemporary local data to inform regional guidelines.

We detected at least one of the four bacterial infections in half of cervicitis cases, which aligns with previous studies (1, 11, 36). While it is possible that a small number of the cervicitis cases without an infection identified had *T. vaginalis* or HSV, our limited data supports their selective testing based on clinical indications for our population. However, guidelines should consider regional risk data as these infections have higher prevalence estimates in other populations. Recent PID studies have reported three broad groups of aetiological organisms; established STIs (*N. gonorrhoeae, C. trachomatis, M. genitalium, T. vaginalis*), BV-associated bacteria (*A. vaginae*, *Sneathia*, *Megasphaera*), and organisms usually associated with the gastrointestinal or respiratory tracts (i.e., *Bacteroides, Escherichia coli, Streptococcus, Haemophilus influenzae*) (31). While PID is a distinct syndrome involving endometrial and upper genital tract infection, cervical infection commonly precedes PID, and it is logical to conclude that the spectrum of organisms involved is similar. The recent microbiota studies of women with cervicitis (12) and PID (30, 31) provide insights into likely infectious aetiologies, and prospective studies using whole metagenome sequencing will provide deeper and broader knowledge of the spectrum of infectious causes of cervicitis.

This study was conducted at the largest public Australian sexual health centre and accessed comprehensive clinical and meta-data. As this retrospective audit was of symptomatic cervicitis cases, these findings may not be generalisable to asymptomatic cases. However, the clinical importance of asymptomatic cervicitis is not clear. Longitudinal prospective studies that include asymptomatic women are challenging but may be needed to further interrogate causality. Additionally, our findings may not apply to other sites with differing population prevalence of infections.

## Conclusion

In this study, BV and/or *M. genitalium* were detected in a third of non-chlamydial and non-gonococcal cervicitis cases, and BV was often the sole diagnosis made. *M. genitalium* was detected as the sole infection in 3% of cervicitis cases, and two-thirds of all cases with *M. genitalum* were co-infected with another infection. How co-infections/polymicrobial infections contribute to cervicitis requires further investigation. These findings build on recent case-control studies showing an association between BV (and BV-associated bacteria) and cervicitis and *M. genitalium* and cervicitis. Though prospective studies are needed to inform causality, these are challenging to undertake and unlikely to be rapidly forthcoming, as they require substantial funding and time. In the meantime, although chlamydia and gonorrhoea account for <30% of infections in cervicitis, testing remains universally recommended by global guidelines. In contrast, despite published data supporting an association, recommendations for testing for *M. genitalium,* and particularly BV, are inconsistent and uncommon outside of specialist sexual health services, potentially leading to delays in appropriate treatment and persistence of infection. Given that >60% of cases of cervicitis are not caused by *C. trachomatis* or *N. gonorrhoeae* in the majority of studies it is time to consider assessing women for BV and *M. genitalium* on presentation with cervicitis, as current empiric treatment may be inadequate.

## Data Availability

The dataset presented in this article is not readily available because data cannot be made publicly available in order to protect patient privacy as per the approved ethics requirement. Requests to access a identified dataset should be directed to LV, lenka.vodstrcil@monash.edu.

## References

[1] Brunham RC Paavonen J Stevens CE Kiviat N Kuo CC Critchlow CW Mucopurulent cervicitis–the ignored counterpart in women of urethritis in men. N Engl J Med. (1984) 311(1):1–6. 10.1056/NEJM198407053110101 6427611

[2] Workowski KA Bachmann LH Chan PA Johnston CM Muzny CA Park I Sexually transmitted infections treatment guidelines, 2021. MMWR Recomm Rep. (2021) 70(4):1–187. 10.15585/mmwr.rr7004a1 34292926 PMC8344968

[3] Johnson LF Lewis DA. The effect of genital tract infections on HIV-1 shedding in the genital tract: a systematic review and meta-analysis. Sex Transm Dis. (2008) 35(11):946–59. 10.1097/OLQ.0b013e3181812d15 18685546

[4] Wiesenfeld HC Hillier SL Meyn LA Amortegui AJ Sweet RL. Subclinical pelvic inflammatory disease and infertility. Obstet Gynecol. (2012) 120(1):37–43. 10.1097/AOG.0b013e31825a6bc9 22678036

[5] Soper DE Wiesenfeld HC. The continued challenges in the diagnosis of acute pelvic inflammatory disease: focus on clinically mild disease. J Infect Dis. (2021) 224(12 Suppl 2):S75–9. 10.1093/infdis/jiab158 34396404

[6] Kiviat NB Paavonen JA Wolner-Hanssen P Critchlow CW Stamm WE Douglas J Histopathology of endocervical infection caused by *Chlamydia trachomatis*, herpes simplex virus, trichomonas vaginalis, and *Neisseria gonorrhoeae*. Hum Pathol. (1990) 21(8):831–7. 10.1016/0046-8177(90)90052-7 2387574

[7] Lis R Rowhani-Rahbar A Manhart LE. *Mycoplasma genitalium* infection and female reproductive tract disease: a meta-analysis. Clin Infect Dis. (2015) 61(3):418–26. 10.1093/cid/civ312 25900174

[8] Latimer RL Vodstrcil LA Plummer EL Doyle M Murray GL Fairley CK The clinical indications for testing women for *Mycoplasma genitalium*. Sex Transm Infect. (2022) 98(4):277–85. 10.1136/sextrans-2020-054818 34210839

[9] Keshavarz H Duffy SW Sadeghi-Hassanabadi A Zolghadr Z Oboodi B. Risk factors for and relationship between bacterial vaginosis and cervicitis in a high risk population for cervicitis in southern Iran. Eur J Epidemiol. (2001) 17(1):89–95. 10.1023/A:1010935723248 11523583

[10] Schwebke JR Weiss HL. Interrelationships of bacterial vaginosis and cervical inflammation. Sex Transm Dis. (2002) 29(1):59–64. 10.1097/00007435-200201000-00010 11773880

[11] Marrazzo JM Wiesenfeld HC Murray PJ Busse B Meyn L Krohn M Risk factors for cervicitis among women with bacterial vaginosis. J Infect Dis. (2006) 193(5):617–24. 10.1086/500149 16453256

[12] Plummer EL Vodstrcil LA Danielewski JA Murray GL Doyle ML Latimer RL Vaginal anaerobes are associated with cervicitis: a case-control study. J Infect. (2024) 89(2):106210. 10.1016/j.jinf.2024.106210 38944285

[13] Ong JJ Bourne C Dean JA Ryder N Cornelisse VJ Murray S Australian sexually transmitted infection (STI) management guidelines for use in primary care, 2022 update. Sex Health. (2023) 20(1):1–8. 10.1071/SH22134 36356948

[14] Miranda AE Silveira MFD Pinto VM Alves GC Carvalho NS. [Brazilian protocol for sexually transmitted infections 2020: infections that cause cervicitis]. Epidemiol Serv Saude. (2021) 30(spe1):e2020587. 10.1590/s1679-4974202100008.esp1 33729399

[15] Young C Argáez C. Management and Treatment of Cervicitis: A Review of Clinical Effectiveness and Guidelines. Ottawa, ON: Canadian Agency for Drugs and Technologies in Health (2017). Available online at: https://www.ncbi.nlm.nih.gov/books/NBK525875/2021 30234930

[16] Marrazzo JM Handsfield HH Whittington WL. Predicting chlamydial and gonococcal cervical infection: implications for management of cervicitis. Obstet Gynecol. (2002) 100(3):579–84. 10.1016/s0029-7844(02)02140-3 12220782

[17] Marrazzo JM Martin DH. Management of women with cervicitis. Clin Infect Dis. (2007) 44(Suppl 3):S102–10. 10.1086/511423 17342663

[18] Taylor SN Lensing S Schwebke J Lillis R Mena LA Nelson AL Prevalence and treatment outcome of cervicitis of unknown etiology. Sex Transm Dis. (2013) 40(5):379–85. 10.1097/OLQ.0b013e31828bfcb1 23588127 PMC3868214

[19] Abraham E Fairley CK Denham I Bradshaw CS Farquharson RM Vodstrcil LA Positivity and risk factors for trichomonas vaginalis among women attending a sexual health clinic in Melbourne, 2006 to 2019. Sex Transm Dis. (2022) 49(11):762–8. 10.1097/OLQ.0000000000001690 35948300 PMC9553257

[20] Su J Tan LY Garland SM Tabrizi SN Mokany E Walker S Evaluation of the SpeeDx ResistancePlus MG diagnostic test for *Mycoplasma genitalium* on the applied biosystems 7500 fast qPCR platform. J Clin Microbiol. (2018) 56(1):e01245. 10.1128/JCM.01245-17 29093102 PMC5744202

[21] Tabrizi SN Su J Bradshaw CS Fairley CK Walker S Tan LY Prospective evaluation of ResistancePlus MG, a new multiplex quantitative PCR assay for detection of *Mycoplasma genitalium* and macrolide resistance. J Clin Microbiol. (2017) 55(6):1915–9. 10.1128/JCM.02312-16 28381611 PMC5442548

[22] Amsel R Totten PA Spiegel CA Chen KC Eschenbach D Holmes KK. Nonspecific vaginitis. Diagnostic criteria and microbial and epidemiologic associations. Am J Med. (1983) 74(1):14–22. 10.1016/0002-9343(83)91112-9 6600371

[23] Nugent RP Krohn MA Hillier SL. Reliability of diagnosing bacterial vaginosis is improved by a standardized method of gram stain interpretation. J Clin Microbiol. (1991) 29(2):297–301. 10.1128/jcm.29.2.297-301.1991 1706728 PMC269757

[24] Durukan D Fairley CK Bradshaw CS Read TRH Druce J Catton M Increasing proportion of herpes simplex virus type 1 among women and men diagnosed with first-episode anogenital herpes: a retrospective observational study over 14 years in Melbourne, Australia. Sex Transm Infect. (2019) 95(4):307–13. 10.1136/sextrans-2018-053830 30554143

[25] Engel JL Fairley CK Greaves KE Vodstrcil LA Ong JJ Bradshaw CS Patterns of sexual practices, sexually transmitted infections and other genital infections in women who have sex with women only (WSWO), women who have sex with men only (WSMO) and women who have sex with men and women (WSMW): findings from a sexual health clinic in Melbourne, Australia, 2011–2019. Arch Sex Behav. (2022) 51(5):2651–65. 10.1007/s10508-022-02311-w 35776396 PMC9293838

[26] Chow EPF Hocking JS Ong JJ Phillips TR Fairley CK. Sexually transmitted infection diagnoses and access to a sexual health service before and after the national lockdown for COVID-19 in Melbourne, Australia. Open Forum Infect Dis. (2021) 8(1):ofaa536. 10.1093/ofid/ofaa536 33506064 PMC7665697

[27] Ling Z Liu X Chen X Zhu H Nelson KE Xia Y Diversity of cervicovaginal microbiota associated with female lower genital tract infections. Microb Ecol. (2011) 61(3):704–14. 10.1007/s00248-011-9813-z 21287345

[28] Gorgos LM Sycuro LK Srinivasan S Fiedler TL Morgan MT Balkus JE Relationship of specific bacteria in the cervical and vaginal microbiotas with cervicitis. Sex Transm Dis. (2015) 42(9):475–81. 10.1097/OLQ.0000000000000318 26267872 PMC4590771

[29] Turpin R Tuddenham S He X Klebanoff MA Ghanem KG Brotman RM. Bacterial vaginosis and behavioral factors associated with incident pelvic inflammatory disease in the longitudinal study of vaginal flora. J Infect Dis. (2021) 224(12 Suppl 2):S137–44. 10.1093/infdis/jiab103 34396403 PMC8499701

[30] Hillier SL Bernstein KT Aral S. A review of the challenges and complexities in the diagnosis, etiology, epidemiology, and pathogenesis of pelvic inflammatory disease. J Infect Dis. (2021) 224(12 Suppl 2):S23–8. 10.1093/infdis/jiab116 34396398 PMC8365114

[31] Mitchell CM Anyalechi GE Cohen CR Haggerty CL Manhart LE Hillier SL. Etiology and diagnosis of pelvic inflammatory disease: looking beyond gonorrhea and chlamydia. J Infect Dis. (2021) 224(12 Suppl 2):S29–35. 10.1093/infdis/jiab067 34396407 PMC8365120

[32] Wiesenfeld HC Meyn LA Darville T Macio IS Hillier SL. A randomized controlled trial of ceftriaxone and doxycycline, with or without metronidazole, for the treatment of acute pelvic inflammatory disease. Clin Infect Dis. (2021) 72(7):1181–9. 10.1093/cid/ciaa101 32052831 PMC8028096

[33] Salado-Rasmussen K Tolstrup J Sedeh FB Larsen HK Unemo M Jensen JS. Clinical importance of superior sensitivity of the aptima TMA-based assays for *Mycoplasma genitalium* detection. J Clin Microbiol. (2022) 60(4):e0236921. 10.1128/jcm.02369-21 35317613 PMC9020335

[34] Bjartling C Osser S Persson K. *Mycoplasma genitalium* in cervicitis and pelvic inflammatory disease among women at a gynecologic outpatient service. Am J Obstet Gynecol. (2012) 206(6):476.e1–8. 10.1016/j.ajog.2012.02.036 22483084

[35] Lokken EM Balkus JE Kiarie J Hughes JP Jaoko W Totten PA Association of recent bacterial vaginosis with acquisition of *Mycoplasma genitalium*. Am J Epidemiol. (2017) 186(2):194–201. 10.1093/aje/kwx043 28472225 PMC5860020

[36] Marrazzo JM. Mucopurulent cervicitis: no longer ignored, but still misunderstood. Infect Dis Clin North Am. (2005) 19(2):333–49. 10.1016/j.idc.2005.03.009 15963875

[37] Currie MJ Bowden FJ. The importance of chlamydial infections in obstetrics and gynaecology: an update. Aust N Z J Obstet Gynaecol. (2007) 47(1):2–8. 10.1111/j.1479-828X.2006.00670.x 17261092

[38] Lusk MJ Garden FL Rawlinson WD Naing ZW Cumming RG Konecny P. Cervicitis aetiology and case definition: a study in Australian women attending sexually transmitted infection clinics. Sex Transm Infect. (2016) 92(3):175–81. 10.1136/sextrans-2015-052332 26586777

[39] Chow EP Fehler G Read TR Tabrizi SN Hocking JS Denham I Gonorrhoea notifications and nucleic acid amplification testing in a very low-prevalence Australian female population. Med J Aust. (2015) 202(6):321–3. 10.5694/mja14.00780 25832159

