# Supplementary material for: Trends in infections detected in women with cervicitis over a decade
Source: Front Reprod Health. 2025 Feb 3;7:1539186. doi: 10.3389/frph.2025.1539186 (PMC11830735; doi:10.3389/frph.2025.1539186)
Supplement: Supplementary file 1 [file Datasheet1.docx]

***Supplementary Material***

*Expanded Methods*

*Testing for genital infections*

*C. trachomatis* was tested for in symptomatic women and *universally screened* for among all women attending MSHC, throughout the study period. Prior to March 2015, this was with a Strand displacement amplification assay (Becton RD BD ProbeTec, Becton Dickinson, Sparks, MD, USA) and subsequently, with a transcription-mediated amplification (TMA) assay (Aptima® Combo-2 [AC2] assay, Hologic Gen-Probe Panther system; Hologic, San Diego, CA, USA). Due to the very low prevalence of *N. gonorrhoeae* in women in Melbourne prior to March 2015(1), indications for *N. gonorrhoeae* testing only included cervicitis, pelvic inflammatory disease (PID), post-coital bleeding, test-of-cure, sex worker, and sexual contact of *N. gonorrhoeae* infection. From March 2011 to March 2015, *N. gonorrhoeae* testing involved culture (swabs plated at the bedside onto a modified Thayer-Martin medium) and a cervical smear that was Gram stained and underwent immediate microscopy. From March 2015, all women were *universally* *screened* for *N. gonorrhoeae* using the multiplex AC2-assay (Hologic, combined with chlamydia). Throughout the study period, *M. genitalium* was not screened for and testing was in accordance with national guidelines and the following indications: cervicitis, pelvic inflammatory disease (PID), post-coital bleeding, test-of-cure, and sexual contact of *M. genitalium* infection. During the study period, *M. genitalium* was tested for using a real-time (q) polymerase chain reaction (PCR) assay targeting the MgPa gene(2) until November 2015. After November 2015, the assay changed to the ResistancePlus™ MG assay (SpeeDx Pty Ltd, Sydney, Australia)(3,4). Vaginal microscopy (Gram stain and wet preparation) was performed in all women presenting with abnormal vaginal discharge, itch, vaginal malodour, pelvic pain, cervicitis, and post-coital bleeding. BV was assessed using both Amsel and Nugent criteria, with the presence of 3 or 4 Amsel criteria and a Nugent score of 4-10 used to diagnose BV(5,6). *T. vaginalis* is extremely uncommon in women in Melbourne(7) and screening asymptomatic infections is not recommended. However, selective testing for *T. vaginalis* and for genital HSV infections (HSV-1, HSV-2) occurred in women with cervicitis based on the presence of relevant clinical signs, risk and indications (i.e. HSV observation of herpetic lesions). In October of 2018, testing for *T. vaginalis* changed from wet preparation and culture to the Aptima® *T. vaginalis* assay (Hologic). Testing for HSV was conducted using an in-house qPCR assay targeting the glycoprotein B gene(8).

**References**

1. Chow EP, Fehler G, Read TR, Tabrizi SN, Hocking JS, Denham I, et al. Gonorrhoea notifications and nucleic acid amplification testing in a very low-prevalence Australian female population. Med J Aust. 2015;202(6):321-3.

2. Jensen JS, Uldum SA, Sondergard-Andersen J, Vuust J, Lind K. Polymerase chain reaction for detection of Mycoplasma genitalium in clinical samples. J Clin Microbiol. 1991;29(1):46-50.

3. Su J, Tan LY, Garland SM, Tabrizi SN, Mokany E, Walker S, et al. Evaluation of the SpeeDx ResistancePlus MG Diagnostic Test for Mycoplasma genitalium on the Applied Biosystems 7500 Fast qPCR Platform. J Clin Microbiol. 2018;56(1):e01245.

4. Tabrizi SN, Su J, Bradshaw CS, Fairley CK, Walker S, Tan LY, et al. Prospective Evaluation of ResistancePlus MG, a New Multiplex Quantitative PCR Assay for Detection of Mycoplasma genitalium and Macrolide Resistance. J Clin Microbiol. 2017;55(6):1915-9.

5. Amsel R, Totten PA, Spiegel CA, Chen KC, Eschenbach D, Holmes KK. Nonspecific vaginitis. Diagnostic criteria and microbial and epidemiologic associations. Am J Med. 1983;74(1):14-22.

6. Nugent RP, Krohn MA, Hillier SL. Reliability of diagnosing bacterial vaginosis is improved by a standardized method of gram stain interpretation. J Clin Microbiol. 1991;29(2):297-301.

7. Abraham E, Fairley CK, Denham I, Bradshaw CS, Farquharson RM, Vodstrcil LA, et al. Positivity and Risk Factors for Trichomonas vaginalis Among Women Attending a Sexual Health Clinic in Melbourne, 2006 to 2019. Sex Transm Dis. 2022;49(11):762-8.

8. Durukan D, Fairley CK, Bradshaw CS, Read TRH, Druce J, Catton M, et al. Increasing proportion of herpes simplex virus type 1 among women and men diagnosed with first-episode anogenital herpes: a retrospective observational study over 14 years in Melbourne, Australia. Sex Transm Infect. 2019;95(4):307-13.
