# Supplementary material for: Trends in infections detected in women with cervicitis over a decade
Source: Front Reprod Health. 2025 Feb 3;7:1539186. doi: 10.3389/frph.2025.1539186 (PMC11830735; doi:10.3389/frph.2025.1539186)
Supplement: Supplementary file 2 [file Table1.docx]

**Supplementary Table 1. Summary of existing cervicitis guidelines.**

| **Guideline name, country and/or body** | **Listed causes and/or recommended testing** | **Treatment recommendations** |
| --- | --- | --- |
| Australia: Australasian Sexual Health Alliance ^1^ | **Causes:** Main causes chlamydia and gonorrhoea  Less common causes *Mycoplasma genitalium, Trichomonas vaginalis,* Herpes Simplex Virus (HSV) | **Empiric treatment:** Doxycycline 100mg 7d bd |
| BMJ Best Practice guidelines ^2^ | **Etiology:** *Neisseria gonorrhoeae* and *Chlamydia trachomatis* listed as most commonly isolated organisms, but states that in most cases no organism is identified.  Vaginal swabs may reveal *T. vaginalis*, HSV type 2, *M. genitalium*, *Gardnerella vaginalis* [marker of bacterial vaginosis], or one of various streptococcal species.  **Testing:** A cervical discharge specimen should be sent for:   - Thayer-Martin culture: a sensitive test for gonococcal infection - Nucleic acid amplification testing: this is the definitive test for detection of *C. trachomatis*, *N. gonorrhoeae*, or *M. genitalium* - Gram stain: gold standard for diagnosis of bacterial vaginosis (BV) - Plus wet mount wet mount examination of cervical discharge, *T. vaginalis* rapid test | **Empiric treatment:** High-risk patients, presumptive treatment to cover chlamydia and gonorrhoea.  Treatment based on CDC guidelines (See below).  **Etiologic treatment:** Treatment is only provided to patients at lower risk of STIs once infection is confirmed by diagnostic testing. |
| Brazil: Revista da Sociedade Brasileira de Medicina Tropical  Brazilian Protocol for Sexually Transmitted Infections, 2020: infections that cause cervicitis ^3^ | **Causes:** The most common agents are *C. trachomatis* and *N. gonorrhoeae*. However, *T. vaginalis*, *M. genitalium*, *U. urealiticum*, and HSV can also cause cervicitis  **Testing:** If evidence of cervicitis, test and treat *C. trachomatis* and *N. gonorrhoeae*  If vaginal discharge, then test for *T. vaginalis* and BV | Treatment is stated to follow laboratory investigations (etiologic treatment) |
| Canada: Canadian Agency for Drugs and Technologies in Health ^4^ | **Causes:** Main causes of cervicitis are chlamydia and gonorrhoea.  Potential causes: *M. genitalium* and BV | **Empiric treatment:** Treatment for chlamydia and gonorrhoea commonly provided |
| European guidelines ^5-7^ | Cervicitis is listed as an indication for testing within each pathogen-specific guideline (*C. trachomatis*, *N. gonorrhoeae* and *M. genitalium*). | **Etiologic treatment:** following laboratory investigations  **Empiric treatment:** If there is evidence of mucopurulent cervicitis in women when rapid test results are not available, and dependent on local gonorrhoea incidence, empirical treatment covering *C. trachomatis* infection, or combined treatment for chlamydial infection and gonorrhoea, should be considered. |
| Germany: “Continuing Medical Education” ^8^ | **Causes:** The main pathogens are *C. trachomatis* and/or *N. gonorrhoeae*; rarer ones include *T. vaginalis* and HSV-2. Limited data suggest that *M. genitalium* can also cause cervicitis. Most cases, no pathogen isolated. | **Empiric treatment:** Azithromycin 1 g oral single dose  Or in case of likelihood of *N. gonorrhoeae:* Ceftriaxone 1 g IM plus 1.5 g azithromycin 1.5g oral single dose  Treat for additional pathogens if detected |
| United Kingdom: British Association for Sexual Health and HIV (BASSH) ^9-12^ | **Causes:** Within “urethritis and cervicitis” tab on BASSH website the following STIs are listed: *C. trachomatis*, *N. gonorrhoeae*, *M. genitalium*  Within each STI-specific publication, cervicitis is listed as associated syndrome.  In the guidelines on “STIs in primary care”:  **Testing:** If there is evidence of cervicitis, consider STIs – take endocervical swabs for *C. trachomatis* and *N. gonorrhoeae*  If trichomoniasis is suspected: *T. vaginalis* is difficult to diagnose in general practice, consider referral. (Diagnose with GUM / laboratory)  If evidence of BV: BV may co-exist with other causes of vaginal discharge (*T. vaginalis*, candida, cervicitis) | Treatment is separately listed under each individual STI |
| United States of America: Centers for Disease Control and Prevention | **Causes:** Most common causes chlamydia and gonorrhoea  Associations with cervicitis: trichomoniasis, genital herpes (especially primary HSV-2 infection), or *M. genitalium* have been associated with cervicitis.  Limited data indicate that BV and frequent douching might cause cervicitis.  **Testing guidelines:**   - Test for chlamydia and gonorrhoea - Evaluate for concomitant BV and trichomoniasis - Consider *M. genitalium* in absence of other causes | **Empiric treatment:** Doxycycline 100 mg orally 2 times/day for 7 days  Consider concurrent treatment for gonococcal infection if the patient is at risk for gonorrhoea or lives in a community where the prevalence of gonorrhoea is high  Alternative Regimen: Azithromycin 1 g orally in a single dose  Trichomoniasis and BV should be treated if detected |
| World Health Organisation (WHO): Guidelines for the management of symptomatic sexually transmitted infections ^13^ | Molecular testing has greatly improved the detection of *C. trachomatis* and *N. gonorrhoeae* among both symptomatic and asymptomatic women and has become the recommended gold standard technology to diagnose and screen populations for these infections.  In settings with limited access to a laboratory: WHO suggests performing a speculum examination and treating for *N. gonorrhoeae* and *C. trachomatis* if there is evidence of cervicitis, and, if available, performing a rapid test for these two infections (and treating based on results).  If vaginal discharge detected, WHO guidelines suggest testing for *T. vaginalis* and BV. But does not mention their independent association with cervicitis.  Does not specifically outline testing for *M. genitalium,* however states that treatment of cervicitis using doxycycline and azithromycin can simultaneously treat *C. trachomatis* and *M. genitalium*. | **Empiric treatment:** If a rapid test is not available, WHO suggests treating people who have signs of cervicitis on speculum examination for infection with *N. gonorrhoeae* and *C. trachomatis*.  **Etiologic treatment:** Treat *T. vaginalis* or BV if detected. |

Key: g, gram; GUM, genitourinary medicine; IM, intramuscular

We searched Ovid MEDLINE and Pubmed databases using a search strategy that included the terms “cervicitis” AND “guideline”, and reviewed reference lists for additional testing and treatment guidelines for cervicitis. All guidelines listed *Chlamydia trachomatis* and *Neisseria gonorrhoeae* as the most common causes of cervicitis, whereas *Mycoplasma genitalium* and bacterial vaginosis were named as potential causes in seven and two guidelines, respectively. All nine guidelines recommended routine testing for *C. trachomatis* and *N. gonorrhoeae*. Only two guidelines explicitly included *M. genitalium* in their testing recommendation, others suggested testing in absence of other causes. Six guidelines included BV in testing recommendations, especially if a vaginal discharge was present. Treatment recommendations also differed, and only three recommended etiologic treatment after laboratory investigations.

**References**

1. Ong JJ, Bourne C, Dean JA, et al. Australian sexually transmitted infection (STI) management guidelines for use in primary care 2022 update. *Sex Health* 2023; **20**(1): 1-8.

2. BMJ Best Practice - Cervicitis. Author: M J Solnik. available here: <https://bestpractice.bmj.com/topics/en-gb/662>, 2022.

3. Miranda AE, Silveira MFD, Pinto VM, Alves GC, Carvalho NS. Brazilian Protocol for Sexually Transmitted Infections, 2020: infections that cause cervicitis. *Rev Soc Bras Med Trop* 2021; **54**(suppl 1): e2020587.

4. Young C, Argaez C. Management and Treatment of Cervicitis: A Review of Clinical Effectiveness and Guidelines. Ottawa (ON); 2017.

5. Lanjouw E, Ouburg S, de Vries HJ, Stary A, Radcliffe K, Unemo M. 2015 European guideline on the management of Chlamydia trachomatis infections. *Int J STD AIDS* 2016; **27**(5): 333-48.

6. Unemo M, Ross J, Serwin AB, Gomberg M, Cusini M, Jensen JS. 2020 European guideline for the diagnosis and treatment of gonorrhoea in adults. *Int J STD AIDS* 2020: 956462420949126.

7. Jensen JS, Cusini M, Gomberg M, Moi H, Wilson J, Unemo M. 2021 European guideline on the management of Mycoplasma genitalium infections. *J Eur Acad Dermatol Venereol* 2022; **36**(5): 641-50.

8. Wagenlehner FM, Brockmeyer NH, Discher T, Friese K, Wichelhaus TA. The Presentation, Diagnosis, and Treatment of Sexually Transmitted Infections. *Dtsch Arztebl Int* 2016; **113**(1-02): 11-22.

9. Nwokolo NC, Dragovic B, Patel S, Tong CY, Barker G, Radcliffe K. 2015 UK national guideline for the management of infection with Chlamydia trachomatis. *Int J STD AIDS* 2016; **27**(4): 251-67.

10. Fifer H, Saunders J, Soni S, Sadiq ST, FitzGerald M. 2018 UK national guideline for the management of infection with Neisseria gonorrhoeae. *Int J STD AIDS* 2020; **31**(1): 4-15.

11. Soni S, Horner P, Rayment M, et al. British Association for Sexual Health and HIV national guideline for the management of infection with Mycoplasma genitalium (2018). *Int J STD AIDS* 2019; **30**(10): 938-50.

12. Sexually Transmitted Infections in Primary Care (RCGP/BASHH) by Lazaro N. available at [www.rcgp.org](https://alfredhealthconnect-my.sharepoint.com/personal/lvodstrcil_mshc_org_au/Documents/Cervicitis/Submitted%20to%20Frontiers%20-%20call%20out/www.rcgp.org) and [www.bashh.org/guidelines](https://alfredhealthconnect-my.sharepoint.com/personal/lvodstrcil_mshc_org_au/Documents/Cervicitis/Submitted%20to%20Frontiers%20-%20call%20out/www.bashh.org/guidelines), 2013.

13. Guidelines for the management of symptomatic sexually transmitted infections. Geneva: World Health Organization. Licence: CC BY-NC-SA 3.0 IGO, 2021.
