# Supplementary material for: Trends in infections detected in women with cervicitis over a decade
Source: Front Reprod Health. 2025 Feb 3;7:1539186. doi: 10.3389/frph.2025.1539186 (PMC11830735; doi:10.3389/frph.2025.1539186)
Supplement: Supplementary file 3 [file Image1.pdf]

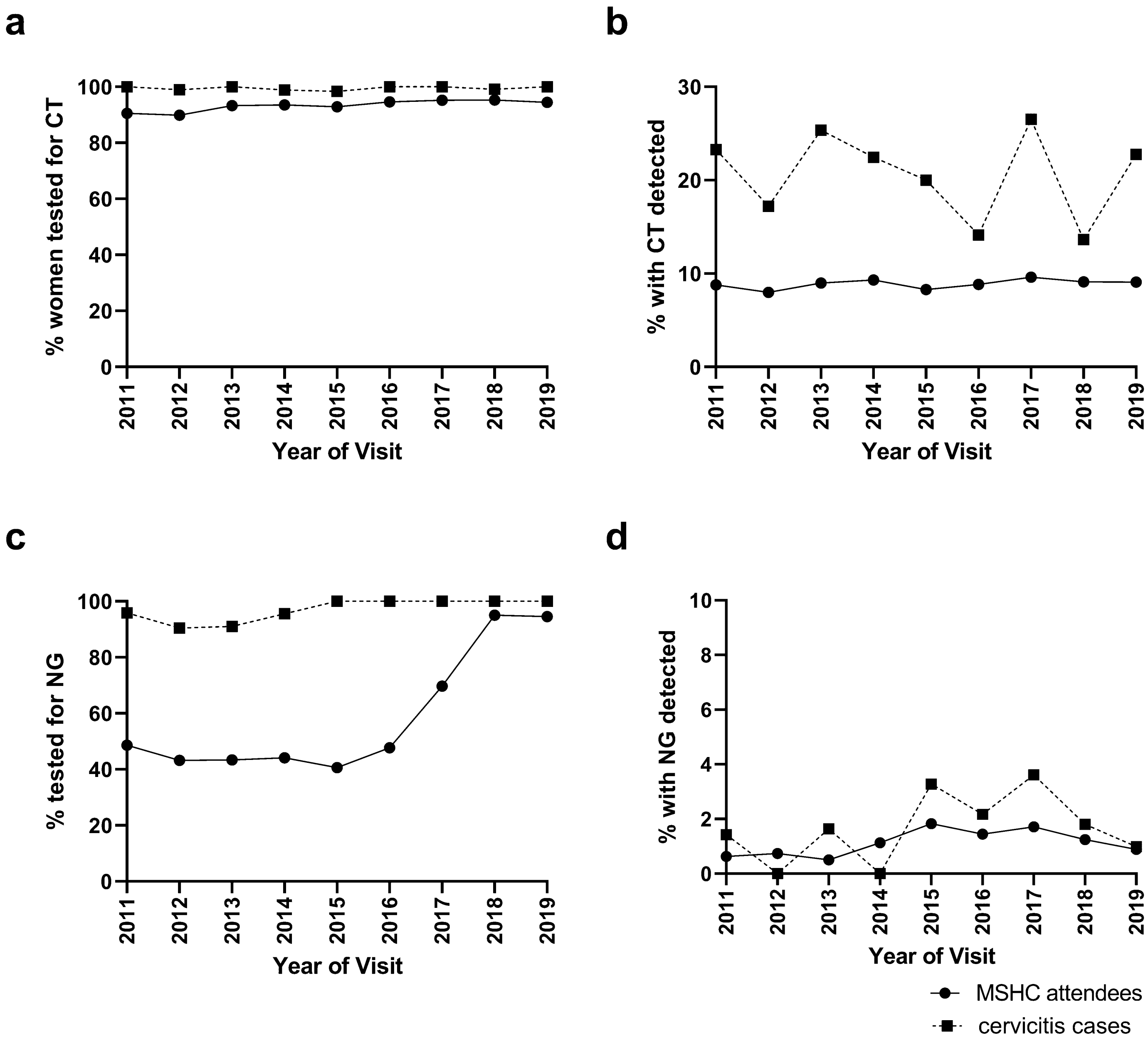

S3. *Chlamydia trachomatis* (CT) and *Neiseria gonorrhoeae* (NG) testing and positivity among women with cervicitis and all female MSHC attendees overtime. The proportion of cervicitis cases or all MSHC first-ever attendees who were (A) tested for *C. trachomatis*, (B) had *C. trachomatis* detected, (C) tested for *N. gonorrhoeae* or (D) had *N. gonorrhoeae* detected at MSHC between 2011 – 2019, was calculated.
